# Design and Experiment of FBG-Based Icing Monitoring on Overhead Transmission Lines with an Improvement Trial for Windy Weather

**DOI:** 10.3390/s141223954

**Published:** 2014-12-12

**Authors:** Min Zhang, Yimeng Xing, Zhiguo Zhang, Qiguan Chen

**Affiliations:** State Key Laboratory of Information Photonics and Optical Communications, Beijing University of Posts and Telecommunications, Beijing 100876, China; E-Mails: 10274023@bjtu.edu.cn (Y.X.); zhangzhiguo@bupt.edu.cn (Z.Z.); noxinlife@163.com (Q.C.)

**Keywords:** overhead power transmission line, ice monitoring, fiber Bragg grating (FBG), strain sensor

## Abstract

A scheme for monitoring icing on overhead transmission lines with fiber Bragg grating (FBG) strain sensors is designed and evaluated both theoretically and experimentally. The influences of temperature and wind are considered. The results of field experiments using simulated ice loading on windless days indicate that the scheme is capable of monitoring the icing thickness within 0–30 mm with an accuracy of ±1 mm, a load cell error of 0.0308*v*, a repeatability error of 0.3328*v* and a hysteresis error is 0.026%. To improve the measurement during windy weather, a correction factor is added to the effective gravity acceleration, and the absolute FBG strain is replaced by its statistical average.

## 1. Introduction

Frequent, long-duration, and widespread icing load can have disastrous effects on overhead power transmission lines, such as short circuits, line breaks, and even tower collapse. For example, the China Southern Power Grid suffered a severe disaster in 2008 owing to transmission line icing. Therefore, valid methods for monitoring icing can have significant benefits for individual transmission lines and for the energy transport infrastructure as a whole.

Traditional methods of estimating icing conditions include video surveillance, dip-sag monitoring, non-contact infrared measurement, and temperature sensing at the line surface or core [1–3]. These methods generally do not provide accurate icing thickness, and they are confronted with difficulty in power-supply and electromagnetic interference. In recent years, various strain sensors and corresponding schemes have been applied to monitor icing [4–7], among which fiber Bragg grating (FBG) sensing has exhibited a great potential in transmission line monitoring [8]. The FBG sensor has the advantages of light weight, small size, robustness against electromagnetic interference and corrosion, high temperature stability, and low attenuation. Particularly, the FBG sensor is capable of accurate phase discrimination without a special reference point. In addition, it can be integrated with optical fiber-based signal transmission systems and thus is suitable for distributed measurement and grid monitoring.

Combining the features of optical fiber composite ground wires (OPGW) and FBG strain sensors, we designed and implemented an icing monitoring scheme for overhead transmission lines. Theoretical analysis and field experiments were conducted. The influences of wind load and temperature variation were considered.

## 2. Principles of FBG Sensing

**Figure 1.**
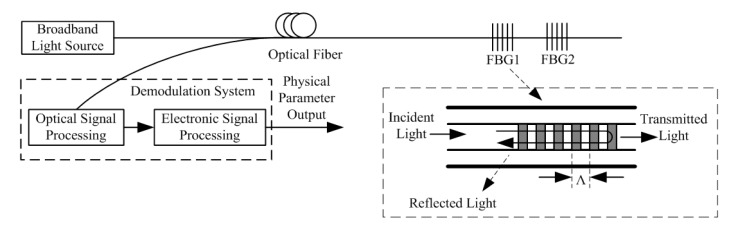
Schematic diagram of the fiber Bragg grating (FBG) sensing system.

A schematic diagram of the FBG sensing system is shown in Figure 1. The FBGs are usually distributed in series along the optical fiber. If a broadband light enters these FBGs, particular wavelengths will be reflected and fed into the demodulation system, whereas the other wavelengths will simply be transmitted. These reflected wavelengths are known as the Bragg wavelengths of the FBGs, defined as [9,10]

(1) $$\lambda_{\text{B}}=2n_{\text{eff}}\cdot \Lambda$$

where *n*_eff_ is the effective index and Λ is the grating period of the FBG.

The parameters neff and Λ are sensitive to external conditions. As a result, the variation in the external conditions can be mapped into a shift in the Bragg wavelength Δλ_B_. As far as the monitoring of icing is concerned, the variations in strain Δε and temperature Δ*T* are relatively more important. The strain-induced Bragg wavelength shift Δλ_Bε_ is approximated by

(2) $$\Delta \lambda_{\text{B}\varepsilon }=\lambda_{\text{B}}(1-P_{\text{e}})\Delta \varepsilon =K_{\varepsilon }\Delta \varepsilon$$

where *P*_e_ is the FBG elasto-optical coefficient and *K*_ε_ is the strain-related sensitivity coefficient.

The temperature-induced Bragg wavelength shift Δλ_BT_ is approximated by

(3) $$\Delta \lambda_{\text{BT}}=(\alpha +\xi )\Delta T=K_{\text{T}}\Delta T$$

where α is the thermal expansion coefficient of FBG, ξ is its thermo-optic coefficient, and *K*_T_ is the temperature-related sensitivity coefficient.

Therefore, according to the measured Δλ_B_ and Δ*T*, we can determine the strain variation Δε. If we relate Δε with the icing load, the monitoring of icing is possible. In our design, the FBG strain sensor serves as a “translator” to relate the icing load with the strain variation.

## 3. Design of the Icing Monitoring System

The proposed icing monitoring scheme consists of a central icing monitoring system and a number of remote sensing units. The icing monitoring system demodulates the sensing signal, displays the information, and alerts operators to possible disaster; the remote sensing units detect and collect the icing thickness along the transmission lines. To fulfill the distributed sensing requirements, these remote sensing units should be implemented together to construct a sensor network [11].

**Figure 2.**
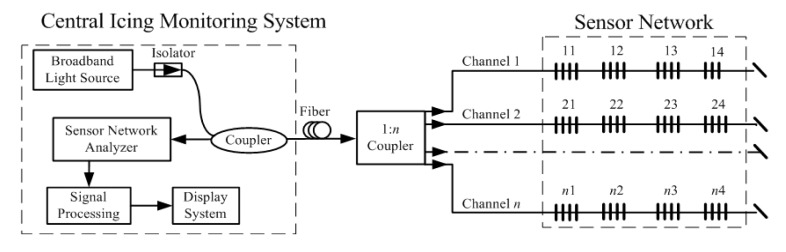
Block diagram of the icing monitoring scheme.

Figure 2 shows a block diagram of the proposed system. The sensor network consists of *n* channels. One of the channels, without an FBG sensor, is used to eliminate the spectral reflectance of the sensor array and thus avoid the reflection interference. Along each of the other *n* − 1 channels, a series of sensing units are distributed. These sensing units can be fixed at a point near the ground wire on the transmission tower. The complete design of an icing monitoring system should include, at a minimum, the strain sensors, wind sensors, and temperature sensors. At the central monitoring system, the optical signal from a broadband light source propagates through an isolator and a coupler and then is divided into *n* parts. These *n* signals enter the sensor network and are reflected at each monitoring point by the FBGs. Then the reflected signals are combined through the coupler and sent to the sensor network analyzer, where they are demodulated and displayed after signal processing.

**Figure 3.**
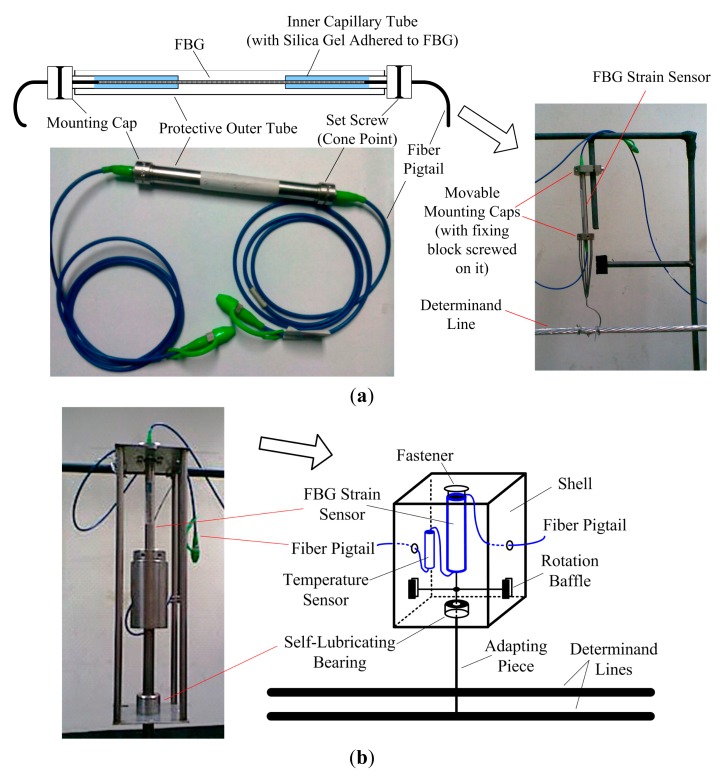
Structures of the FBG strain sensor (**a**) and the remote sensing unit (**b**).

The remote sensing unit and the strain sensor are critical to the icing monitoring scheme. In our design, we choose to use silica-based FBG strain sensors to avoid the temperature influence, because they usually have good thermal stability, such as small thermal expansion coefficient and thermo-optic coefficient, and a compact solid structure. The structure of the FBG strain sensor is shown in Figure 3a. It has tubular encapsulation. In the outer protective steel tube, are the coaxial inner capillary tubes to contain the FBG. The FBG is adhered to the inner tubes and the adhesive is usually made of stress sensitization materials with a small Young's modulus, such as silica gel, epoxide resin, and acrylic polymer. There are two mounting caps at the ends of the inner tubes, from which the fiber pigtails are guided out.

To construct the remote sensing unit, we implemented the strain sensor in a shell, as shown in Figure 3b. To prevent the sensor from swinging in the wind, and to keep it vertical to the ground, we fixed the sensor's upper mounting cap onto the shell's cover and guided its lower mounting cap out of the shell through a narrow hole by means of an adapting piece with a self-lubricating bearing. In addition, to prevent the sensor from rotating, we implemented a pair of rotation baffles in the shell to fix the sensor further. The other end of the adapting piece is connected to the determinand line. The adapting piece with the self-lubricating bearing and the rotation baffles are designed to keep the FBG disalignment-proof and thus improve the sensing accuracy. Additionally, we connected a temperature sensor in series to the FBG strain sensor to monitor the ambient temperature. The whole unit was tightly packaged, waterproof, and corrosion-resistant.

The icing load and the weights of the determinand line and the adapting piece cause deformational stress in the adhesive and then yield axial strain in the FBG. To demodulate the strain caused by icing load, the weights of the determinand line and adapting piece, and the influence of temperature variance, should be excluded.

The icing load and the weights of the determinand line and the adapting piece cause deformational stress in the adhesive and then yield axial strain in the FBG. To demodulate the strain caused by icing load, the weights of the determinand line and adapting piece, and the influence of temperature variance, should be excluded.

Assume that the length, mass, and diameter of the determinand line are *L*, *m*, and *d*, respectively. The mass of the adapting piece is *m*_1_, the icing thickness is *h*, and the icing load is *m*_ice_. The internal FBG sensor can be viewed as a spring with an elastic coefficient of *E*. We have

(4) $$\left(m+m_{1}+m_{\text{ice}}\right)g=E\cdot \Delta x$$

where Δ*x* is the spring deformation and *m*_ice_ is determined by

(5) $$m_{\text{ice}}=\rho \pi \left[{\left(\frac{d}{2}+h\right)}^{2}-{\left(\frac{d}{2}\right)}^{2}\right]L$$

where ρ is the icing density.

Without consideration of temperature influence, assuming that Δλ_B_ is proportional to the external force and the strain is proportional to λ_B_, we have

(6) $$\Delta \lambda_{\text{B}}=k_{1}\left(m+m_{1}+m_{\text{ice}}\right)g$$

(7) $$\varepsilon =k_{2}\Delta \lambda_{\text{B}}$$

where *k*_1_ and *k*_2_ are the linear coefficients.

Solving Equations (5)–(7) simultaneously, we obtain the expression of icing thickness as

(8) $$h=\sqrt{\frac{1}{\rho \pi L}\left(\frac{\varepsilon }{k_{1}k_{2}g}-m-m_{1}\right)+\frac{d^{2}}{4}}-\frac{d}{2}$$

Equation (8) indicates that, if *L*, *m*, *d*, *m*_1_, *k*_1_, and *k*_2_ are known, the icing thickness *h* can be calculated with measured FGB strain ε. This method is simple and practical.

If the temperature compensation is considered, the Bragg wavelength shift becomes

(9) $$\Delta \lambda_{\text{B}}=\Delta \lambda_{\text{B}\varepsilon }+\Delta \lambda_{\text{BT}}$$

and Equation (7) should be rewritten as

(10) $$\varepsilon =k_{2}\Delta \lambda_{\text{B}}=k_{2}k_{1}\left(m+m_{1}+m_{\text{ice}}\right)g+k_{2}K_{\text{T}}\Delta T$$

Substituting Equation (8) into Equation (10), we have

(11) $$h=\sqrt{\frac{1}{\rho \pi L}\left(\frac{\varepsilon -k_{2}K_{\text{T}}\Delta T}{k_{1}k_{2}g}-m-m_{1}\right)+\frac{d^{2}}{4}}-\frac{d}{2}$$

## 4. Experimental Results and Discussion

**Figure 4.**
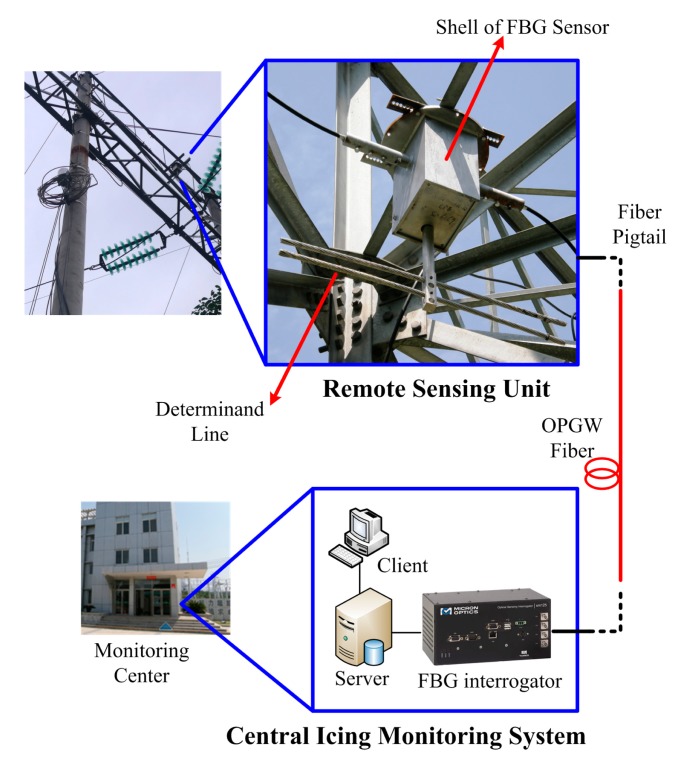
Implemented icing monitoring system in the experiments.

The reported research has proven that a wire in unit length, namely the determinand line, can replace the real OPGW in validating the monitoring of icing, only if the determinand line is the same as the OPGW except for the length, and if both lines are located at the same electric tower [12]. Therefore, in our experiments, we implemented the remote sensing unit onto an electric tower and applied a determinand line as the OPGW equivalent around that tower. The implemented icing monitoring system and the surroundings of the experiments are shown in Figure 4.

The determinand line used is OPGW-2S 1/24B1 (64/46–93.4) by ZhongTian Tech. Ltd. (Nantong, China), a type of OPGW widely used in Chinese power systems. The diameter *d* = 14.00 mm, the length *L* = 1.00 m, and the weight *m* = 0.497 kg. The bearing area per unit length is 110.3 mm^2^, the nominal tensile strength is 78.8 kN, the Young's modulus is 105.4 kN/mm^2^, and the thermal expansion coefficient is 16.6 × 10^−6^/°C. Additionally, the weight of the adapting piece *m*_1_ is 1.23 kg.

The FBG strain sensor used is BGK-FBG-4000 by BGeokon Ltd. (Beijing, China) and its main parameters are as follows: The center wavelength is 1561.405 nm, the strain measuring range is ±15,000 με, the operating temperature range is within −20 and +80 °C, the scale distance is 150 mm, the strain distinguishability is 1 με, the strain coefficient is 785.63271154 με/nm, and the linear coefficients satisfy *k*_1_*k*_2_ = 74.22601 με/kg. The FBG interrogator in the central icing monitoring system is an SM-125 by Micron Optics Int. with an operating wavelength range of 1510–1590 nm, scanning frequency of 2 Hz, and a precision is 1 pm.

**Figure 5.**
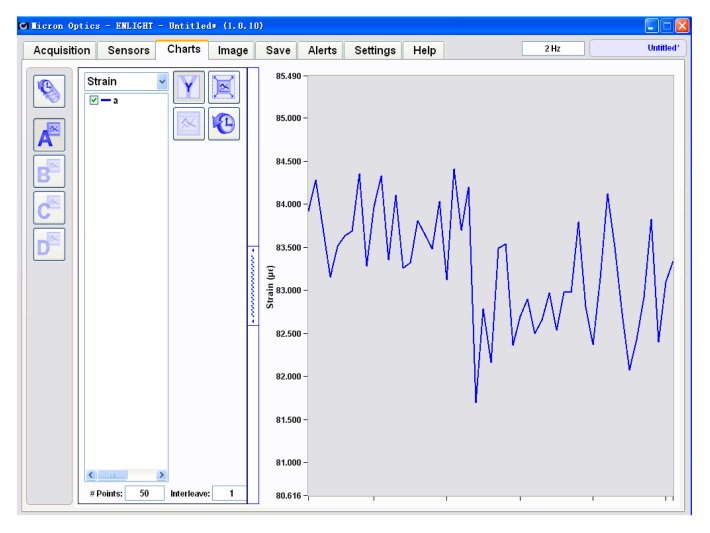
Variation in FBG strain in the unloading experiment.

The selection of the strain sensor depends on the requirement of icing monitoring. If the required icing thickness precision is 1 mm, that is, a precision of icing load of 40 g per unit length, the strain distinguishability is not necessarily as fine as 1 με. We conducted an unloading experiment before the loading experiment. The variation in the FBG strain in the unloading experiment is provided in Figure 5. The yielded strain shows a random vibration within 3 με. For an icing thickness precision of 1 mm, the corresponding FBG strain is about 9 με. Therefore, a strain distinguishability of 5 με is sufficient.

With respect to the hysteresis quality of the strain sensor, the sampling time interval in our design is 0.5 s, whereas the response time of silica FBG strain sensor is usually on the order of 10^−7^ s. Specifically, for strain sensing, the response time of a tube-encapsulated silica FBG sensor is typically 2.1 × 10^−7^ s. Thus, the dynamic response of the silica FBG strain sensor is far faster than the sampling frequency and therefore the hysteresis effect can be neglected.

**Figure 6.**
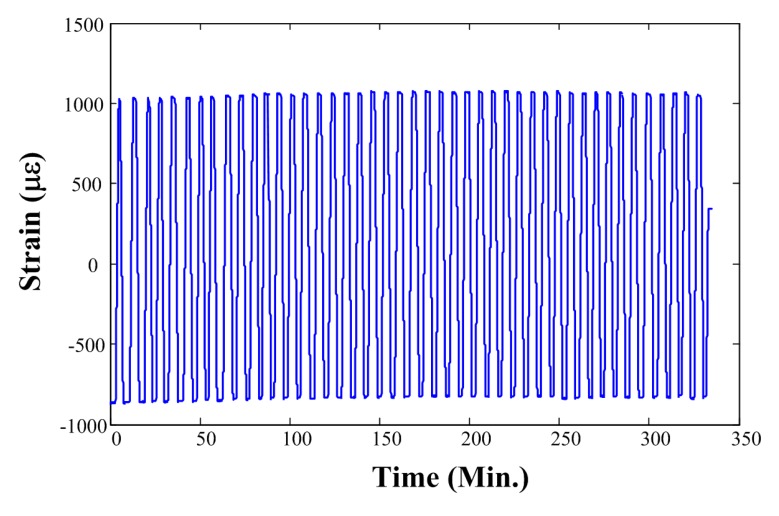
Strain in repeated experiments with alternative forces of 20 kN and 45 kN.

We also tested the system stability through repeated loading experiments. We exerted two vertical forces of 20 kN and 45 kN, alternatively, on the determinand line. Each turn took 1 min. The recorded strain is shown in Figure 6, where the strain rises very slowly at the beginning because of the inelastic deformation of the newly produced OPGW. The strain tends to level off after about 20 turns. The results show that the strain sensor is relatively stable during repeated stretches and relaxations.

Temperature-related stability is also important to the strain sensor, because it has to be used in harsh outdoor environments. The polymer optical fiber (POF) sensor is a commonly used optical fiber sensor. Rapid progress has been made in recent years in the properties of POF sensors [13–16]. POF sensors now have lower attenuation, better durability, and wider measuring range [15,17]. In comparison with silica fiber sensors, POF sensors usually have higher coupling efficiency, smaller Young's moduli, and smaller elasto-optical coefficients. When used as a strain sensor, after removing the load, the POF can return to its original shape quickly. Therefore, POF strain sensors can be utilized in the monitoring of icing. However, POF sensors generally have bad thermal stability [16,18]. Their thermal expansion coefficients and thermo-optic coefficients are generally two orders of magnitude higher than those of the silica fiber sensors. Moreover, their reflected wavelengths are susceptible to temperature effects. Specifically, their Bragg wavelengths usually shift nonlinearly with temperature variation. To apply a POF sensor in harsh environments, such as for monitoring icing, its thermal stability needs to be strengthened.

**Figure 7.**
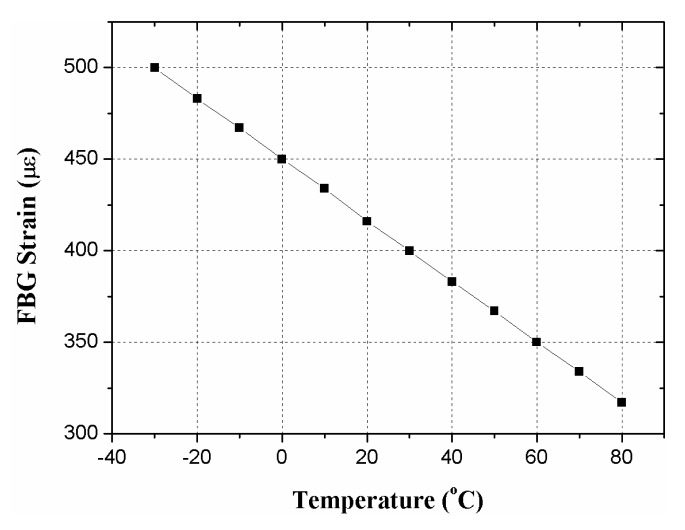
Theoretical FBG strain as a function of temperature with the same load.

Before the loading experiment, we evaluated the strain variation with temperature, as plotted in Figure 7. It shows that the FBG strain decreases about 1.66 με per °C increase in temperature. For example, with the same load, as the temperature rises from −30 °C to 80 °C, the FBG strain drops about 183 με. Accordingly, to obtain accurate strain sensing in harsh outdoor environments, we connected an FBG temperature sensor, the os4200 by Micron Optics Inc. (Atlanta, GA, USA.), to the strain sensor in series and considered the temperature compensation in FBG demodulation.

**Figure 8.**
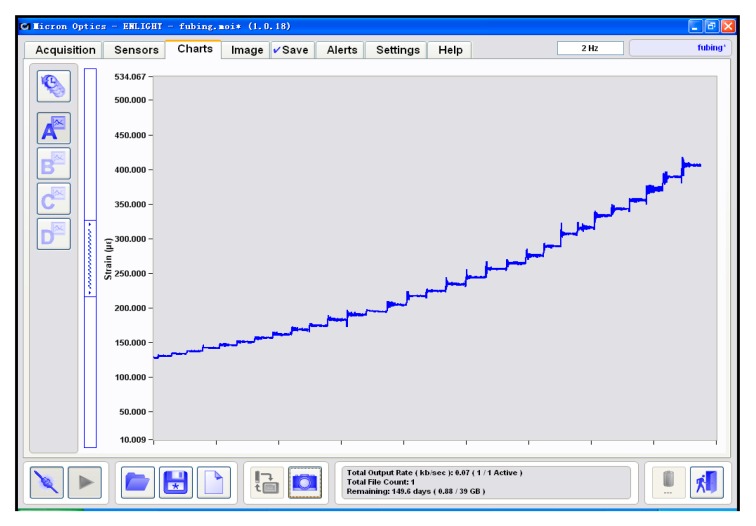
Statistical average of FBG strain by the interrogator as the icing thickness rises.

**Figure 9.**
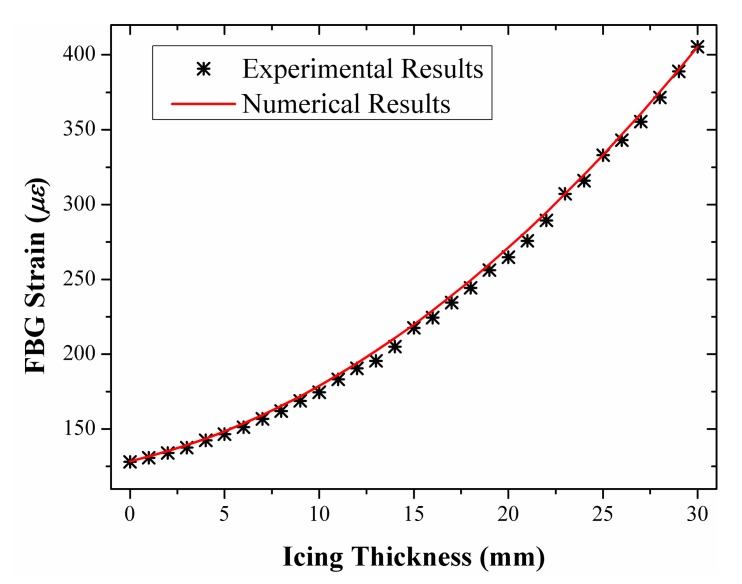
Comparison of experimental and simulation results of FBG strain as a function of icing thickness.

The experiments were carried out by increasing the pensile weights step by step in order to imitate the icing process. The icing thickness was monitored distributedly and the statistical averages of the experimental results were calculated. Specifically, the results of the average FBG strain displayed at the FBG interrogator are shown in Figure 8. We exported the results from the FBG interrogator and compared them with the theoretical results, as shown in Figure 9. The experimental results match with the theoretical results to a large degree and the maximum relative absolute error is only 2.54%.

According to the metrological regulations in standard OIML R60 [19] and its corresponding national standard of China JJG669-2003 [20], we calculated the key parameters of the FBG strain sensor, including sensitivity, load cell error, repeatability error, hysteresis error and creep. During the measurements, we at first got a reference strain indication at 20 °C. Then we repeated the operations at 30 °C, 10 °C and then at 20 °C again. We set the five target strains as 130 με, 300 με, 700 με, 1100 με and 1500 με. At a certain temperature, with the load increased step by step up to its maximum we recorded the measured strains and then we measured the strain again with the load decreased to its minimum. We repeated the operations above 3 times.

The load cell error *E*_L_ was calculated as the difference between the average strain and the reference indication for each test load at each temperature. *E*_L_ equals 0.0308*v* where *v* refers to the load cell verification interval. An ideal estimation of *v* is 1 με, namely, the distinguishability of the strain sensor according to its manual, while the conservative value of *v* is 10 με according to the unloading experiment as shown in Figure 5.

With the same loading and environmental conditions, we made three repeatable tests and calculated the maximum difference among the test results. The calculated repeatability error *E*_R_ is 0.3328*v*.

The hysteresis error was obtained according to the difference between the measured strains for the same applied load. One reading was obtained by increasing the load from its minimum and the other by decreasing the load from its maximum. The result of the hysteresis error is 0.026%, *i.e.*, 0.7801/3000, where 0.7801 με is the maximum difference and 3000 με is the full scale of the sensor.

Applying the constant maximum test load, we calculated the creep as the greatest difference between the initial indication obtained at the test load after stabilization period and any indication obtained over 30 min test period. The calculated creep *C*_C_ is 0.0104*v*.

## 5. Correction Factor for Wind

The experimental results were usually correct according to the pensile weights added in calm and gentle breeze conditions. However, when the wind force was stronger, the yielded results showed apparent dependence on the wind speed and differed from the actual weight.

**Figure 10.**
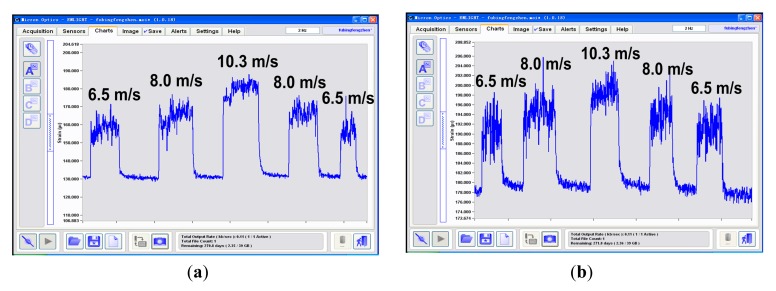
FBG strains measured at different wind speeds, with artificial icing thickness of 3 mm (**a**) and 10 mm (**b**).

To illustrate the influence of the wind on the scheme, we measured the strains with different loadings and wind speeds. In the experiment, we gave the determinand line known loads and then provided an artificial wind perpendicular to the line by turning on and off an air blower. The results are shown in Figure 10, where the strain vibrates apparently in the wind and the extra strain induced by wind pressure is also easy to identify. For example, If the icing thickness is 3 mm, the corresponding strain should be appoximately 128 με according to Figure 9. However, with a wind perpendicular to the line, the actually measured strains are increased by about 26 με, 35 με and 50 με at the wind speed of 6.5 m/s, 8.0 m/s and 10.3 m/s respectively, as shown in Figure 10a. If the icing thickness increases, the extra strains at the same wind speeds become relatively smaller, specifically about 13 με at 6.5 m/s, 16 με at 8.0 m/s and 21 με at 10.3 m/s respectively, as shown in Figure 10b. It indicates that with heavier loading the strain tends to be insensitive to the wind speed.

**Figure 11.**
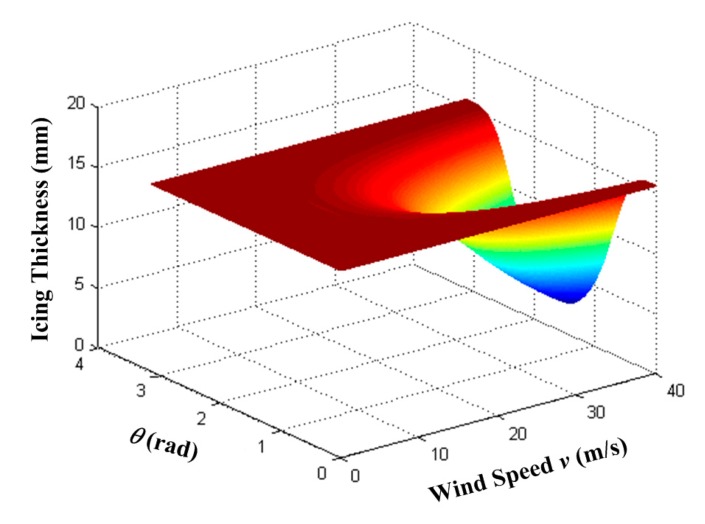
Icing thickness as functions of the wind speed *v*, and the angle between wind direction and determinand line θ.

We analyzed theoretically the wind influence on the monitoring of icing. With the parameters of the determinand line described in Section 4, the icing thickness is plotted as functions of *v* (the wind speed) and θ (the angle between wind direction and determinand line), as shown in Figure 11. If θ approaches to 0 or π, or if *v* is slower than 5 m/s, the wind load exerts very limited influence on the monitoring of icing. However, as θ approaches to π/2 and *v* is faster than 10 m/s, the icing thickness appears to drop. Therefore, the wind load should be considered in the monitoring of icing if a strong wind blows perpendicularly to the determinand line.

According to Figure 10, the stronger the wind, the larger the error introduced in the measurement. To solve this problem, we added a correction factor to the FBG demodulation equations, namely, Equations (8) and (11).

If the wind force is weaker than Beaufort scale 5, we still apply Equation (8) or Equation (11) in demodulation. If the wind force is equal or stronger than Beaufort scale 5, we apply Equation (12) by replacing *g* in Equation (8) or Equation (11) with a modified effective acceleration of gravity *g*_2_. For example, Equation (11) is modified as

(12) $$h=\sqrt{\frac{1}{\rho \pi L}\left(\frac{\varepsilon -k_{2}K_{\text{T}}\Delta T}{k_{1}k_{2}g_{2}}-m-m_{1}\right)+\frac{d^{2}}{4}}-\frac{d}{2}$$

The expression of *g*_2_ is given as

(13) $$g_{2}=\sqrt{g^{2}+\Delta g_{w}^{2}}$$

where Δ*g*_wind_ is the wind-related correction factor and its empirical equation is given as follows [21,22]

(14) $$\Delta g_{\text{wind}}=g_{1}\times \frac{\alpha C_{\text{shape}}\left(d+2h\right)v^{2}}{m+m_{1}}k\sin^{2}\theta \times 10^{-3}$$

where *k* is the wind pressure coefficient in kN/m^2^, α is the non-uniformity coefficient of the wind speed, and *C*_shape_ is the shape factor of wind load and given as follows [22]

(15) $$C_{\text{shape}}=\{\begin{array}{c} \begin{array}{cc} 1.2 & \text{if}d+2h<17\text{mm} \end{array}\\ \begin{array}{cc} 1.1 & \text{if}d+2h\geq 17\text{mm} \end{array} \end{array}$$

The values of α with various wind speeds are given in Table 1 for 330-kV transmission lines and in Table 2 for 500-kV transmission lines [22].

In addition to the wind pressure discussed above, the wind vibration should also be considered. In practice, the quantity and direction of the wind speed may change abruptly from time to time, which causes fluctuation of the FBG strain. To avoid the influence of wind vibration, we took measures in terms of mechanism and signal processing. Mechanically, we applied the adapting piece with self-lubricating bearing and the rotation baffles to keep the FBG disalignment-proof. In signal processing, we took the moving average filter among neighboring samples during a time interval in order to smooth over the fast fluctuation of the measured strain. For example, if during time interval Δ*t*_1_ we obtain *n* samples of ε, *i.e.*, ε_11_, ε_12_,…, ε_1_*_n_*, we take their average *ε̄*_1_ as the yielded FBG strain. We calculate the icing thickness for that time interval with ε in Equation (12) replaced by *ε̄*_1_.

**Figure 12.**
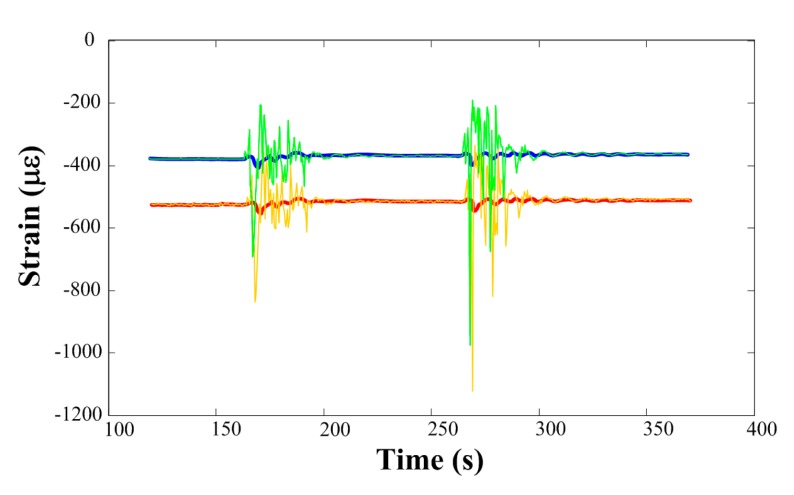
Strain fluctuation due to artificial wind vibration and results of the moving-average filter (green and blue curves: strain and its processed result without temperature compensation; orange and red curves: strain and its processed result with temperature compensation).

We tested the method of the moving-average filter in an experiment with simulated wind vibration. We gave the determinand line a known load and then implemented an artificial wind vibration by turning on and off an air blower. The measured strain is shown in Figure 12, where the green curve and the orange curve are the strain without and with temperature compensation, respectively. The fluctuations of these two curves are due to the artificial wind vibration. We used the moving-average filter in signal processing during the demodulation and the results are given by the blue curve and the red curve, respectively. According to the known load on the determinand line, these processed results match with the actual situation to a large degree.

With the modified demodulation approach, we conducted an experiment on a windy day. The average wind speed during that period was about 16.5 m/s and the gust velocity was beyond 20 m/s. The mass of the pensile weights was 5 kg. The other conditions were the same as given in Section 4.

**Figure 13.**
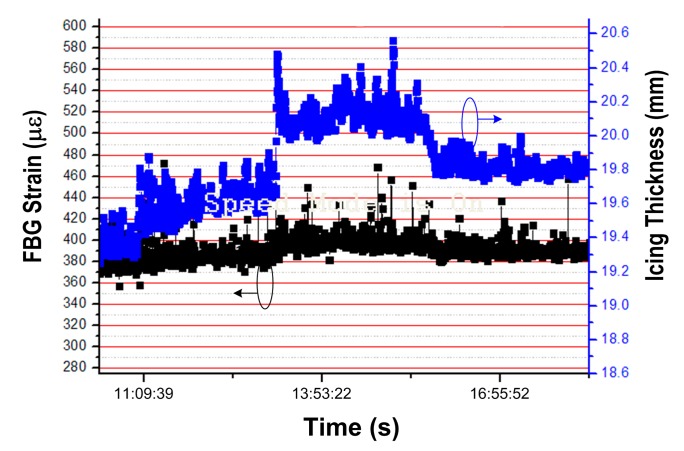
Experimental results of FBG strain and icing thickness through modified demodulation approach with consideration of the wind.

The obtained results in Figure 13 are generally in accordance with what we predicted. By comparison with the results on windless day in Figure 9, the strain in Figure 13 is apparently higher. Specifically, for the same icing thickness of 20.2 mm, the strain for a windy day is about 400 με in Figure 13, and it is only about 265 με for windless day in Figure 9. We believe these results are more reasonable for the windy situation, because the extra FBG strain of about 135 με is caused by the wind pressure.

**Figure 14.**
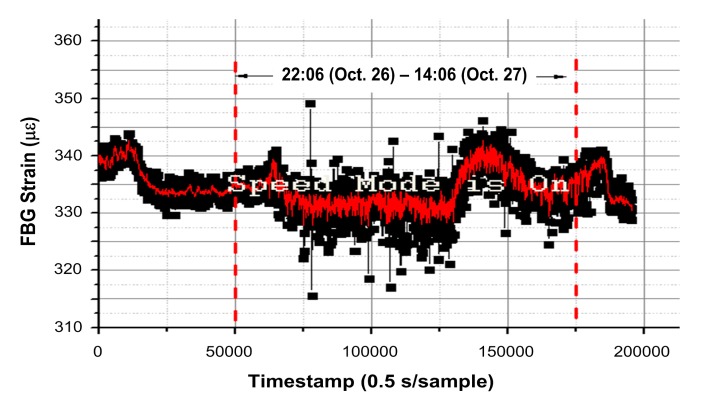
Experimental results of FBG strain with consideration of the wind and in case of no ice coating.

As a comparison with Figure 13, we added the measured FBG strain under normal condition (without icing load) through simulated field experiments during 22:06, 26 October–14:06, 27 October, as shown in Figure 14, during which period the average wind speed was 15.8 m/s and the gust velocity was beyond 20 m/s. The average strain was approximately 334 με under the wind pressure, which is 206 με larger than it in windless day (*i.e.*, 128 με in Figure 9). Therefore, by comparison of the extra strain of 135 με with icing load of 5 kg in Figure 13, the strain tends to be more sensitive to the wind pressure with no or thinner ice coating.

## 6. Conclusions and Future Work

We have implemented and demonstrated a scheme for monitoring icing on overhead transmission lines with an FBG strain sensor. The numerical results and the simulated field experimental results match with each other to a large degree. The minimum variation in the icing thickness that can be identified is 1 mm. The load cell error *E*_L_ is 0.0308*v*, the repeatability error *E*_R_ is 0.3328*v* and the hysteresis error is 0.026%. Experiments on windy days facilitated the improvement of the FBG demodulation method by adding a correction factor to the effective gravity acceleration and replacing the absolute strain with a statistical average.

Long-term field experiments with different conductors, in snowy days and windy days are to be conducted in the future to evaluate the scheme more rigorously. Additionally, our icing monitoring approach is a type of weighting method, which requires the strain sensor to be perpendicular to the ground. For unbalanced icing, Equation (4) is not suitable and the sensing error increases. With the weighting method, the unbalanced load will inevitably induce error. A better approach may be the method of pendency, which works whether the strain sensor is vertical or not.

## Figures and Tables

**Table 1.** Non-uniformity coefficient of wind speed (α) for 330-kV transmission lines.

**Wind speed***v***(m/s)**	**<20**	**20–30**	**30–35**	**>35**
Non-uniformity coefficient α	1.0	0.85	0.75	0.7

**Table 2.** Non-uniformity coefficient of wind speed (α) for 500-kV transmission lines.

**Wind speed***v***(m/s)**	**<10**	**10–20**	**>20**
Non-uniformity coefficient α	1.0	0.75	0.61
